# Using ESPEN data for evidence-based control of neglected tropical diseases in sub-Saharan Africa: A comprehensive model-based geostatistical analysis of soil-transmitted helminths

**DOI:** 10.1371/journal.pntd.0012782

**Published:** 2025-01-09

**Authors:** Jessie Jane Khaki, Mark Minnery, Emanuele Giorgi

**Affiliations:** 1 The Centre for Health Informatics, Computing, and Statistics (CHICAS), Lancaster Medical School, Lancaster University, Lancaster, United Kingdom; 2 Malawi Liverpool Wellcome (MLW) Programme, Blantyre, Malawi; 3 School of Global and Public Health, Kamuzu University of Health Sciences, Blantyre, Malawi; 4 Evidence Action, Deworm the World Initiative, Washington DC, United States of America; IRCCS Sacro Cuore Don Calabria Hospital, ITALY

## Abstract

**Background:**

The Expanded Special Project for the Elimination of Neglected Tropical Diseases (ESPEN) was launched in 2019 by the World Health Organization and African nations to combat Neglected Tropical Diseases (NTDs), including Soil-transmitted helminths (STH), which still affect over 1.5 billion people globally. In this study, we present a comprehensive geostatistical analysis of publicly available STH survey data from ESPEN to delineate inter-country disparities in STH prevalence and its environmental drivers while highlighting the strengths and limitations that arise from the use of the ESPEN data. To achieve this, we also propose the use of calibration validation methods to assess the suitability of geostatistical models for disease mapping at the national scale.

**Methods:**

We analysed the most recent survey data with at least 50 geo-referenced observations, and modelled each STH species data (hookworm, roundworm, whipworm) separately. Binomial geostatistical models were developed for each country, exploring associations between STH and environmental covariates, and were validated using the non-randomized probability integral transform. We produced pixel-, subnational-, and country-level prevalence maps for successfully calibrated countries. All the results were made publicly available through an R Shiny application.

**Results:**

Among 35 countries with STH data that met our inclusion criteria, the reported data years ranged from 2004 to 2018. Models from 25 countries were found to be well-calibrated. Spatial patterns exhibited significant variation in STH species distribution and heterogeneity in spatial correlation scale (1.14 km to 3,027.44 km) and residual spatial variation variance across countries.

**Conclusion:**

This study highlights the utility of ESPEN data in assessing spatial variations in STH prevalence across countries using model-based geostatistics. Despite the challenges posed by data sparsity which limit the application of geostatistical models, the insights gained remain crucial for directing focused interventions and shaping future STH assessment strategies within national control programs.

## Author summary

The Expanded Special Project for the Elimination of Neglected Tropical Diseases (NTDs, ESPEN) was established in 2019 to help monitor and control NTDs such as Soil-transmitted helminths (STH) in African countries. We carried out a geostatistical analysis of STH data for 35 countries from the ESPEN database. Separate geostatistical models were developed for each country to tailor the selection of spatial covariates and estimation of covariance parameters to the unique spatial patterns across countries. Moreover, it was observed that the geostatistical models exhibited inadequate calibration in some countries, and thus carrying out spatial predictions at unsampled locations was not possible. These findings urge caution in developing an Africa-wide model based solely on ESPEN data, given the observed heterogeneity in the model parameter estimates and the challenges encountered in model calibration across different species and countries. Despite challenges posed by data sparsity, the insights gained remain crucial for directing focused interventions and shaping future STH assessment strategies within national control programs.

## Introduction

Soil-transmitted Helminthiases (STH) are the most common type of Neglected Tropical Diseases (NTDs) and are caused by parasitic worms, including whipworms (*Trichuris trichiura*), hookworms (*Necator americanus* and *Ancylostoma duodenale*), and roundworms (*Ascaris lumbricoides*) [1, 2]. Approximately 24% (1.5 billion) of the global population experiences annual infections of STH, with high prevalences among children and women of reproductive age, who are at the highest risk for morbidity associated with STH. [1–3]. Populations that mostly suffer from STH infections are found in China, sub-Saharan Africa, East Asia, and the Americas [2, 4]. In sub-Saharan Africa, STH affect more than 11% of the population [3]. However, the STH burden greatly varies both between and within each country of the African continent [3, 4]. Although the STH mortality rate is low, STH are associated with both lower health outcomes (such as anemia and malnutrition) and poor cognitive performance [5–7]. One of the interventions for controlling the transmission of STH is mass drug administration (MDA), otherwise known as preventive chemotherapy (PC). The PC drugs are primarily given to preschool and school-age children and pregnant women to contribute to reducing STH-related morbidities. The frequency of the MDA programs is usually determined according to prevalence classes defined by the WHO, namely <2%, 2%-10%, 10%-20%, 20%-50% and >50% [1, 8, 9]. Understanding the level of burden of STH is thus crucial to assist the efficient allocation of drugs.

The Expanded Special Project for the Elimination of Neglected Tropical Diseases (ESPEN) was established in 2016 as a collaborative effort between the World Health Organization (WHO) African region office, African NTD endemic countries and other NTDs partners [10]. The ESPEN was instituted to help mobilize financial, political, and technical resources. ESPEN aims to contribute to mitigating the effects of the 5 most prevalent NTDs in Africa which, in addition to STH, are trachoma, lymphatic filariasis, schistosomiasis, and onchocerciasis. The ESPEN electronic data portal contains publicly available geo-located sub-national prevalence data on the aforementioned high-burden NTDs, as well as Loiasis. The ESPEN portal also provides both spatial and time-referenced information for some countries. Historical applications of ESPEN data have involved the application of geostatistical mapping of diseases such as schistosomiasis, onchocerciasis, and STH at both country and continent (Africa) levels to inform survey designs and strategies for preventive therapy [3, 11–14].

Model-based geostatistics (MBG) has become an established methodology for prevalence mapping and for better understanding the spatial distribution of disease risk [15–17], thus providing valuable insights for guiding interventions, survey designs, and resource allocations [18–21]. MBG methods for global disease mapping has been instrumental in studying disease distribution across Africa; see, for example, the extensive application of MBG from the Institute of Health Metrics (IHME) in the mapping of HIV/AIDS, onchocerciasis, lymphatic filariasis, maternal and child health, and other health-related indicators [12, 22–27]. Several studies have utilized geostatistical methods to map STH and inform interventions by fitting either a single continent-wide model or have limited their analysis to a single country model [3, 28–30].

The view adopted in this study is that developing a single model for the entire African continent might prove unsuitable, given the diverse climatic and geopolitical landscapes across countries which could be excessively complex to fully capture in a single model using spatially sparse survey data. To address the disparities across countries in relation to STH risk, the adoption of a single Africa-wide model needs to carefully consider two fundamental aspects: the extensive use of spatial risk factors that can best capture the environmental and socioeconomic variation across the continent; and the use of complex covariance structure that accounts for non-stationary residual effects. Prior analyses of STH data incorporated a diverse set of covariates, including socio-economic indicators, (e.g. nightlights and gross domestic product), climatic variables (e.g. precipitation and temperature), and environmental variables (e.g. soil components and elevation) [3, 14, 28, 30]. Of the studies that provided details on the type of covariance function used, most have adopted stationary Matérn and exponential correlation functions [14, 28, 29, 31, 32]. Similarly, in the studies carried out by IHME on mapping other health outcomes at the continent level, a stationary Matérn function was adopted and approximated using stochastic partial differential equations [12, 23, 26, 27]. The adoption of a stationary Matérn becomes more justifiable if the study area is relatively small and/or the covariates have allowed us to account for most of the non-stationary effects from the variation of the outcome. In this study, we pursue a simpler modelling approach to global mapping that aims at formulating context-specific geostatistical models tailored to individual countries, thereby enhancing our understanding of soil-transmitted helminths (STH) dynamics and their differences across countries. In contrast to the use of a single African-wide model, we show that this approach allows us to account for the spatially heterogeneous effects of spatial covariates as well as to better understand the differences in the predictive performance of MBG methods across the continent.

Most of the MBG mapping for STH have adopted cross-validation methods to assess the performance of the fitted geostatistical models [3, 14, 29–33]. In these studies, the focus was primarily on quantifying the accuracy and precision of point predictions through receiver operating curves, root mean square error summaries and mean absolute error [3, 14, 29–34]. One of the issues inherent to these cross-validation approaches is that they treat the observed fraction of positive cases as the true disease prevalence against which the model predictions are assessed [35, 36]. This assumption is especially problematic in low-prevalence settings, where the observed fraction is often zero, making it a poor proxy for the true prevalence [36, 37]. Furthermore, commonly used metrics such as mean square error (MSE) focus solely on the accuracy of point estimates, failing to account for the uncertainty in predictions. In geostatistical modeling, uncertainty quantification is crucial, as it reflects the variability and reliability of predictions across the study area, which point-based metrics like MSE cannot capture. In this study, we use an alternative approach that uses the non-random probability integral transform (nrPIT) method originally proposed to calibrate count data models [35]. We show that one of the main advantages of the nrPIT is that it enables us to evaluate the overall consistency between the data and the predictive distribution of prevalence which is essential to establish the reliability of the predictive inferences derived from geostatistical models [36].

The majority of prior studies on the mapping of STH prevalence did not attempt to classify sub-national units according to the WHO STH prevalence classes [28, 31–34], except for Sartorius et al. [3] where a single threshold of 20% prevalence was used for the classification. In this study, we show how geostatistical models can be used to classify sub-national units based on the WHO STH prevalence classes (<2%, 2%-10%, 10%-20%, 20%-50%, and >50%) that are used to inform the frequency of MDA and other interventions.

In summary, the specific objectives of this paper are as follows:

- to demonstrate how to make the best use of publicly available STH survey data from the ESPEN portal;
- to highlight between countries differences in terms of the importance of environmental risk factors and spatial correlation structure in STH prevalence;
- to highlight the limitations of global mapping when using spatially sparse data, through the non-randomized integral probability transform (nrPIT).

## Materials and methods

### Analysis outline

**Fig 1:**
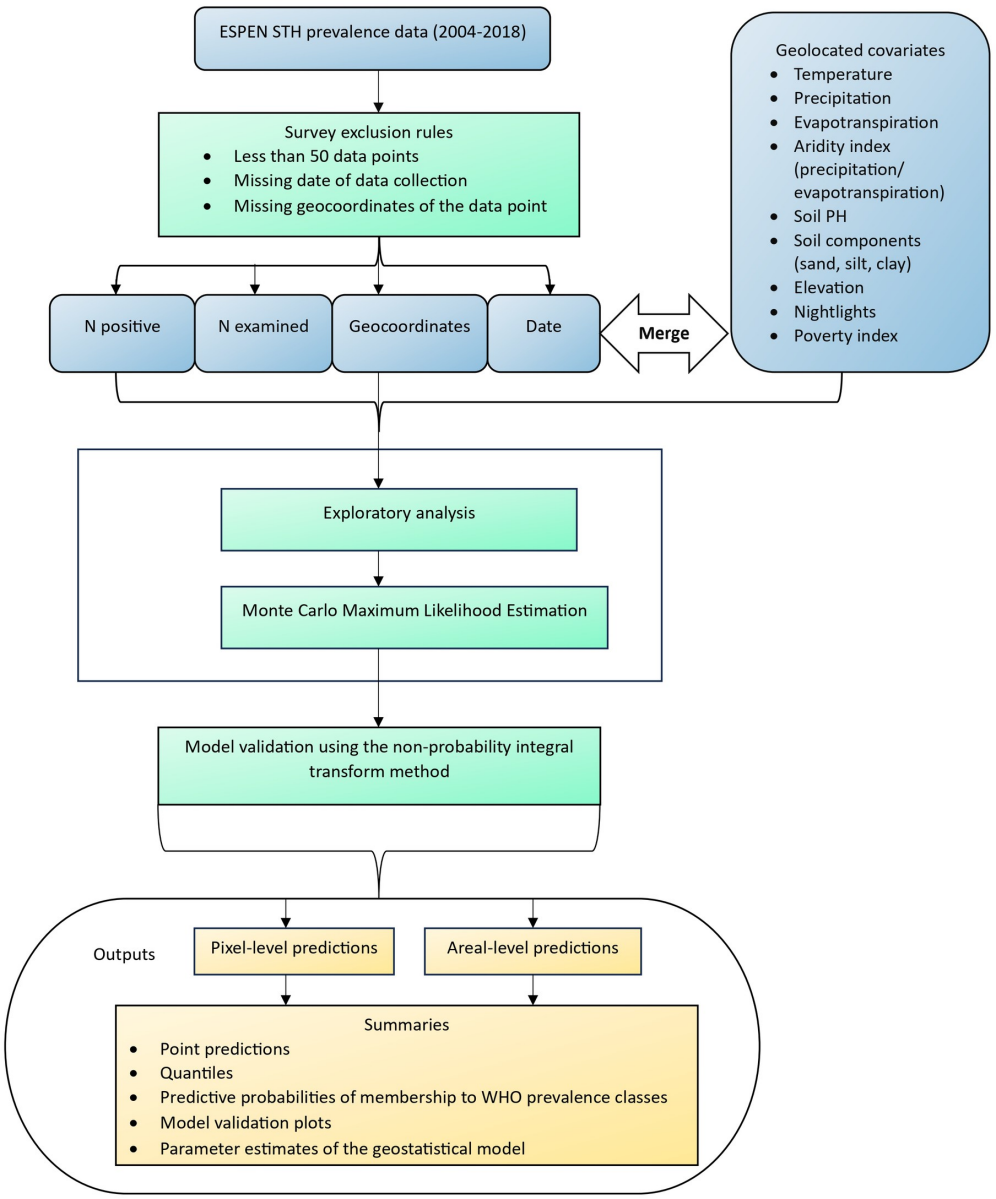
Schematic overview of the modelling and mapping procedures and techniques. The blue boxes denote the input data or materials. The green boxes indicate processes, procedures, and models. The orange boxes describe the output data.

The workflow of the geostatistical analysis is summarised in Fig 1 and consists of the following steps:

1. We extracted the latest STH prevalence data for each country and only considered data-sets that provided information on the year of data collection and geo-referenced coordinates for the sample locations.
2. We extracted climatic and environmental covariates and merged these with the STH prevalence data.
3. We assessed the relationships between covariates and STH prevalence, separately for each species. For countries where prevalence data were not available for each species, we instead used the prevalence of infection with any STH.
4. We tested for residual spatial correlation using the variogram computed on the random effects of a non-spatial Binomial mixed model.
5. The prevalence data were fitted to a Binomial geostatistical model via the Monte Carlo maximum likelihood method.
6. The calibration of the models was validated using the non-randomized probability integral transform.
7. If the model successfully passed the previous validation step, we then used this the generate predictive inferences at country-, sub-national- and pixel-level.

In the following paragraphs, we provide more details for each of the steps outlined above.

### The ESPEN data on STH prevalence

**Fig 2:**
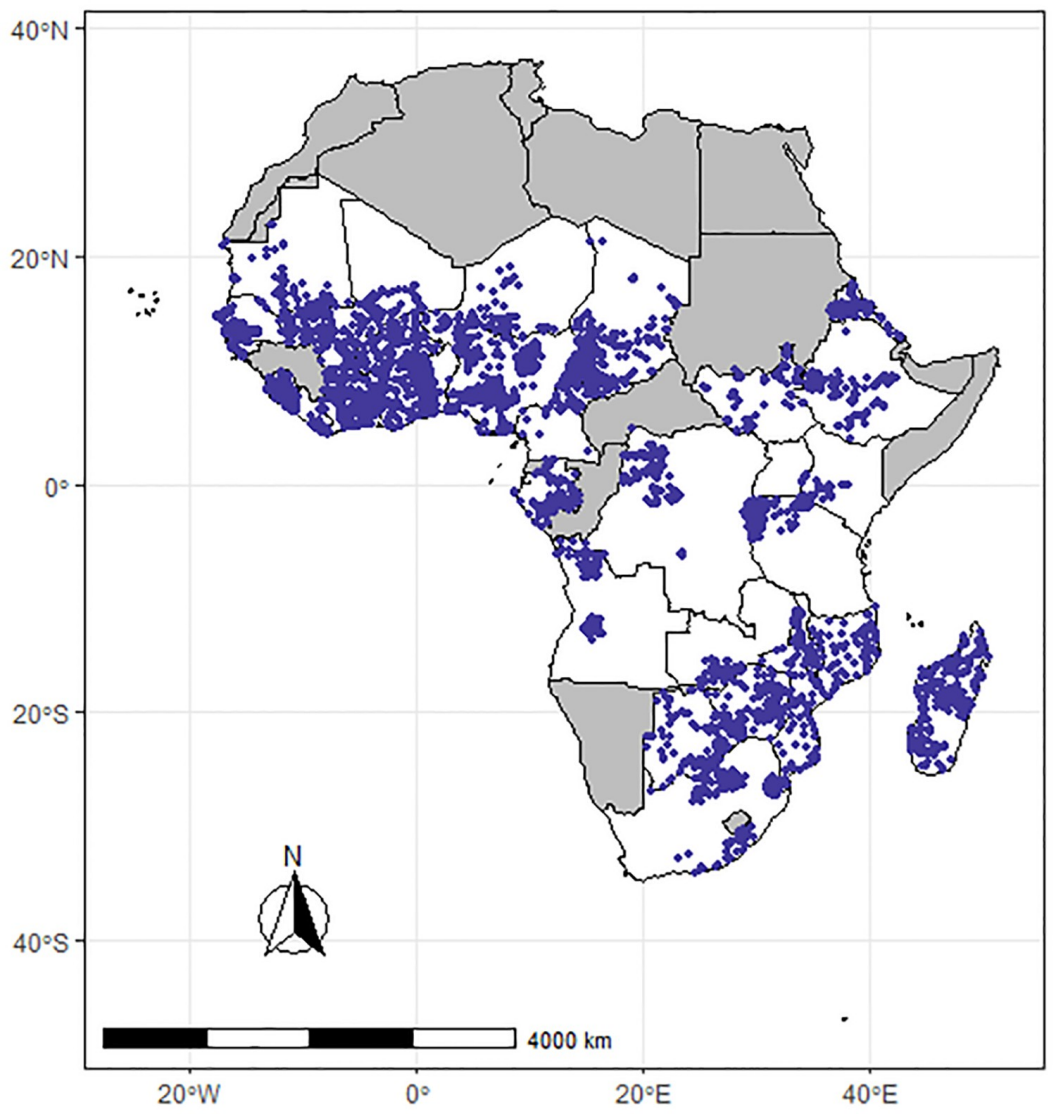
Map illustrating the locations of STH cases. The shaded areas represent countries with no data. The map’s boundaries, names, and designations are derived from Global Administrative Areas (GADM), available at https://gadm.org/ [42]. They do not reflect any opinions of the authors or their affiliated institutions regarding the legal status of any country, territory, city, area, or its authorities, nor the delineation of its borders or boundaries.

The geographical area of interest in this study is the sub-Saharan region. Publicly available geo-referenced prevalence soil-transmitted Helminthiases (STH) survey data were extracted from the Expanded Special Project for Elimination of Neglected (ESPEN) tropical diseases database (https://espen.afro.who.int/). The ESPEN database is a publicly available database that stores data for several neglected tropical diseases. The most recent survey data were retrieved from the website for each country. Full details of data reporting to ESPEN can be found at https://espen.afro.who.int/. Our requirement for inclusion of a country was a sample size of at least 50 observations with complete information on the STH species (hookworm, roundworm, whipworm) or overall STH (any STH), the year of data collection, and geo-coordinates (longitude and latitude). The 50-sample size criterion was based on previous studies showing that small sample sizes of fewer than 50 data points in geostatistical data lead to issues such as overly noisy variograms. Furthermore, in geostatistical studies, small sample sizes (fewer than 50) result in variograms displaying little or no spatial correlation [38–41]. In total, 35 countries complied with this requirement. Fig 2 is a point map illustrating the locations of the observations that were used in the study. For the countries in grey, either the STH data were unavailable, or the sample size was less than 50.

### Climatic and environmental data

Our analysis uses spatially referenced climatic and environmental covariates that have been previously used to map STH prevalence [3]. More precisely, we considered maximum temperature, mean precipitation, and evapotranspiration, which were obtained from TerraClimate database [43]. An aridity index variable was derived by computing the proportion of the precipitation to the evapotranspiration of a country. An increase in the levels of climatic variables such as precipitation and aridity index have been shown in other studies to also increase the prevalence of STH [3]. Previous studies have also shown that the prevalence of STH decreases with an increase in the amount of soil PH and soil texture (clay, sand, silt). We, therefore, extracted covariates on soil acidity and soil texture (clay, sand, and silt) from the International and Soil Reference and Information Centre (ISRIC) [44]. Lastly, we downloaded elevation, nightlight and poverty index data from the Worldpop website [45]. Empirically, it has been found that higher altitudes are generally associated with lower STH risk, especially for *Trichiura* [28]. As expected, it has also been reported that an increase in wealth-related indicators is associated with a decrease in the prevalence of STH [3]. In this study, we used nightlights and poverty indices as proxies for estimating the level of wealth.

The spatial resolution and data sources for the covariates considered in this study are given in Table 1. The geographical locations (longitude and latitude) and year of data collection of the implementation units in the survey data were used to link the survey data to the spatial covariates.

**Table 1:** List of explanatory covariates used in the study and their spatial resolutions.

Name	Spatial resolution	Source
**Soil type and content**		
Soil PH in water	250 m	World Soil Information [44]
Soil type/texture fraction (sand, silt, clay)	250 m	World Soil Information [44]
**Climatic variables**		
Mean precipitation	4 km	TerraClimate [43]
Maximum temperature	4 km	TerraClimate [43]
Potential Evapotranspiration (PET)	4 km	TerraClimate [43]
Aridity index	4 km	Ratio of mean precipitation to PET
**Other variables**		
Elevation	100 m	Worldpop [45]
Nightlights	100 m	Worldpop [45]
Poverty index	1 km	Worldpop [45]

### Data analysis

We first carry out an exploratory analysis to assess the relationship between STH prevalence (species-specific or overall STH) and the spatial covariates. We investigated multicollinearity and chose among highly correlated covariates (those with a correlation surpassing 0.6, following the recommendations and methodologies observed in prior research [46]. To select covariates, we fitted a Binomial generalized linear mixed model where, conditional on mutually-independent distributed Gaussian variables, *Z*_*i*_, the logit linear predictor for prevalence, for a given STH species, is defined as:

$$\begin{array}{c} \text{log}\{\frac{p_{j}(x_{i})}{1-p_{j}(x_{i})}\}=d(x_{i})\beta +Z_{i} \end{array}$$ (1)

where *d* (**x**_*i*_) is the vector explanatory variables to be selected and *β* is a vector of regression coefficients.

The selection of covariates was carried out using a backward stepwise approach, in which the models were compared using the likelihood ratio test. After carrying out the selection of covariates, we tested for residual spatial correlation using the empirical variogram based on the random effects *Z*_*i*_ using a permutation test [36, 47]. If the residual spatial correlation was detected, we then fitted a geostatistical model, which is obtained by introducing a spatial Gaussian process, *S*(**x**_*i*_) and, hence, we modify (1) as:

$$\begin{array}{c} \text{log}\{\frac{p_{j}(x_{i})}{1-p_{j}(x_{i})}\}=d(x_{i})\beta +S(x_{i})+Z_{i} \end{array}$$ (2)

In the above equation, *S* (**x**_*i*_) is a zero-mean stationary and isotropic Gaussian process with an exponential function with variance *σ*^2^, hence

$$\text{Cov}\;\{S\;(x_{i}),S\;(x_{j})\}=\sigma^{2}\;\text{exp}\;\{-u_{ij}/\phi \}$$

where *u*_*ij*_ denotes any distance between any two areas **x**_*i*_ and **x**_*j*_ and *ϕ* is a scale parameter that determines the rate at which the spatial correlation decays to 0 as the distance *u*_*ij*_ increases. The exponential covariance function used in this study is a specific case of the Matérn covariance function, where the parameter *kappa* (*κ*) is set to 0.5 [47].

In countries where species-specific data were available, we fitted model 2 to each of the three species. For Mozambique, Togo, and Zimbabwe only the overall STH prevalence was available, hence, we fitted a single geostatistical model to this outcome. When fitting the model to species separately, we obtained the prevalence of infection with any STH species as:

$$\begin{array}{c} 1-\{\left(1-p_{\text{HK}}\left(x\right)\right)\times \left(1-p_{\text{ASC}}\left(x\right)\right)\times \left(1-p_{\text{TT}}\left(x\right)\right)\} \end{array}$$

where *p*_HK_(*x*), *p*_ASC_(*x*), and *p*_TT_(*x*) are the prevalence for hookworm, *Ascaris* and *Trichiura*, respectively. In the above equation, the expression for the prevalence of any STH species is obtained by assuming that the underlying spatial processes that modulate the three prevalences in the equation are independent conditionally on the spatial covariates used in the models. We point out that this assumption is less strong than the assumption of mutual independence between the three STH species that has been previously made in other studies [28, 48, 49].

The model parameters for Eq 2 were estimated using a Monte Carlo maximum-likelihood (MCML) approach in the PrevMap package in R [50].

### Model validation

To assess the model fit, we used the non-randomized probability integral transform (nrPIT) method that was first proposed for count data models and later adapted to validate binomial geostatistical models [35, 36]. If we let *Y* = {*Y*_*i*_; 1 =, …, *n*} denote the vector of random variables of the number of STH (any STH or species-specific) positive cases; $Y_{i}^{*}$ denote the random variable of the positive tested STH (any STH or species-specific) cases at a set of hold-out locations say $x_{j}^{*}$ for *j* =, …, *q*; and *Q*(*Z*) denote the cumulative density function of a random variable *Z*; the nrPIT is defined as:

$$\begin{array}{c} \text{nrPIT}\left(u\;|\;y_{j}^{*},y\right)=\{\begin{array}{cc} 0 & \text{if}\;u\leq Q(y_{j}^{*}-1\;|\;y)\\ \frac{\left[u-Q\left(y_{j}^{*}-1\;|\;y\right)\right]}{\left[Q\left(y_{j}^{*}\;|\;y\right)-Q\left(y_{j}^{*}-1\;|\;y\right)\right]} & \text{if}\;Q\left(y_{j}^{*}-1\;|\;y\right)\leq u\leq Q\left(y_{j}^{*}\;|\;y\right)\\ 1 & \text{if}\;u\geq Q(y_{j}^{*}\;|\;y) \end{array}. \end{array}$$ (3)

A detailed explanation of the nrPIT can be found in the S1 Text for this paper and other work [35, 36]. Briefly, the nrPIT method uses the following steps:

1. Divide the dataset into a training set and a test set using a random approach.
2. Use the binomial geostatistical models that have been fitted to generate the predictive distribution of prevalence for the locations within the test set.
3. Employ the nrPIT to the positive cases observed in the test set.
4. Evaluate whether the transformed data from the nrPIT method conform to a uniform distribution by analyzing the cumulative density function.

The steps above were implemented for 30%, 40%, and 50% hold-out samples for each model.

For countries and species that validation indicated that the geostatistical models were well calibrated, we then proceeded to carry out predictions as explained in the next section.

### Spatial prediction and policy-relevant criteria for STH interventions

For country and species data-sets analysed, we use the fitted geostatistical models to carry out inferences on the following predictive targets.

1. The **spatially continuous surface** of prevalence defined as:
  $$\begin{array}{c} p(A)=\{p(x):x\in A\} \end{array}$$ (4)
  where *A* denotes the area encompassed by the boundaries of a given country.
2. The **district-level prevalence**, which we define as follows. Let *D*_*k*_ be the set of spatial regions that partition the study country *A* into *k* = 1, 2,.., *K* subunits. Then the predictive target for subunits was defined as:
  $$\begin{array}{c} p\left(D_{k}\right)=\frac{1}{|D_{k}|}\int_{D_{k}}p\left(x\right)dx \end{array}$$ (5)
  where |*D*_*k*_| is the area for subunit *k*. The above integral is approximated using a regular grid covering |*D*_*k*_| with a spatial resolution of 95%. In this study, we used second-level administrative units from the Global Administrative Areas (GADM) website for each country as sub-national boundaries [42].
3. The **country-level prevalence**, which we define as:
  $$\begin{array}{c} p\left(A\right)=\frac{1}{\left|A\right|}\int_{A}p\left(x\right)dx \end{array}$$ (6)
  where *A* represents the area encompassed by the boundaries of a given country, as defined above.

We sample from the joint distribution of prevalence at all pixels and then aggregate according to Eqs 5 and 6 for the administrative-level and country-level predictions.

We obtained 10,000 predictive samples using the Laplace sampling approach implemented in the PrevMap package [50]. For the spatial continuous surface of prevalence, we use a regular grid covering a given country, whose spatial correlation (*ϕ*) is chosen so that the correlation between adjacent pixels is 95% [36, 51].

To classify the districts of a country into predefined classes of prevalence, we compute the predictive probability of falling in any given class based on the fitted models. For this, we use the WHO classification for STH prevalence, namely less than 2%, 2% to 10%, 10% to 20%, 20% to 50%, and greater than 50%. Hence, we allocate a district to one of those classes’ prevalence based on the highest predictive probability.

## Results

A total of 35 countries had STH data with at least 50 observations on the ESPEN database. The year of the last reported data-set on ESPEN varied from 2004 to 2018. About 67% of the data-sets are from 2014 onwards. The number of data points per country ranged from 50 to 1,054, with a median of 129 and an interquartile range of 86 to 265. The list of countries with their sample size and year of data collection can be found in the Shiny applications associated with this paper (Pixel-level results application and Subnational-level and other results application).

In the remainder of this section, we provide a summary of the results at the national level and provide a comprehensive summary of model validation for each country.

Taking Rwanda as a representative case, we further explain how to interpret the findings for each of the 35 countries, which can be accessed using the Shiny application at the links Pixel-level results application and Subnational-level and other results application.

### Country-level results

#### Country-level predictions

**Fig 3:**
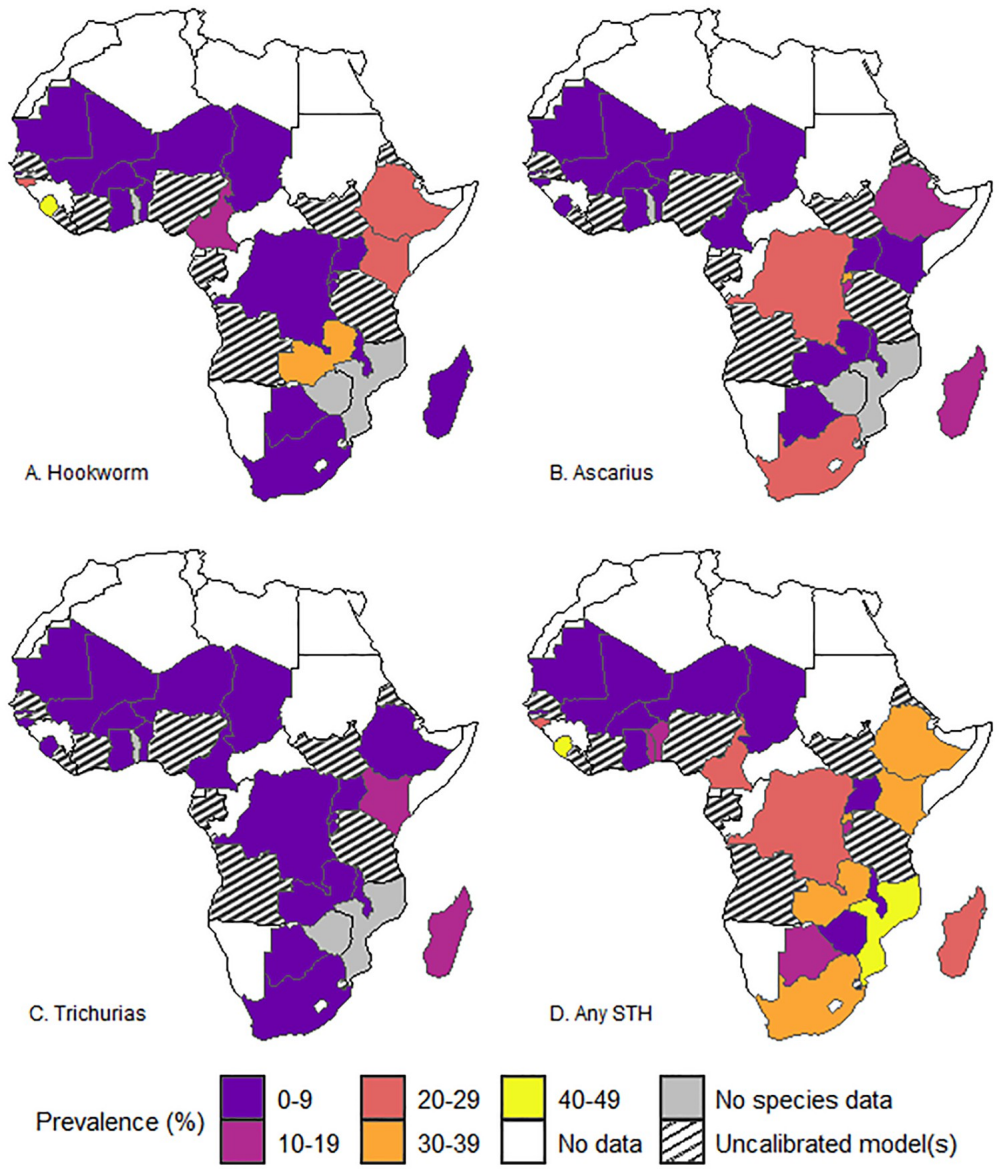
Map showing the country-level predicted geographic distribution of Hookworm (A), *Ascaris* (B), *Trichiura* (C), and overall STH (D). The map’s boundaries, names, and designations are derived from Global Administrative Areas (GADM), available at https://gadm.org/ [42]. They do not reflect any opinions of the authors or their affiliated institutions regarding the legal status of any country, territory, city, area, or its authorities, nor the delineation of its borders or boundaries.

**Fig 4:**
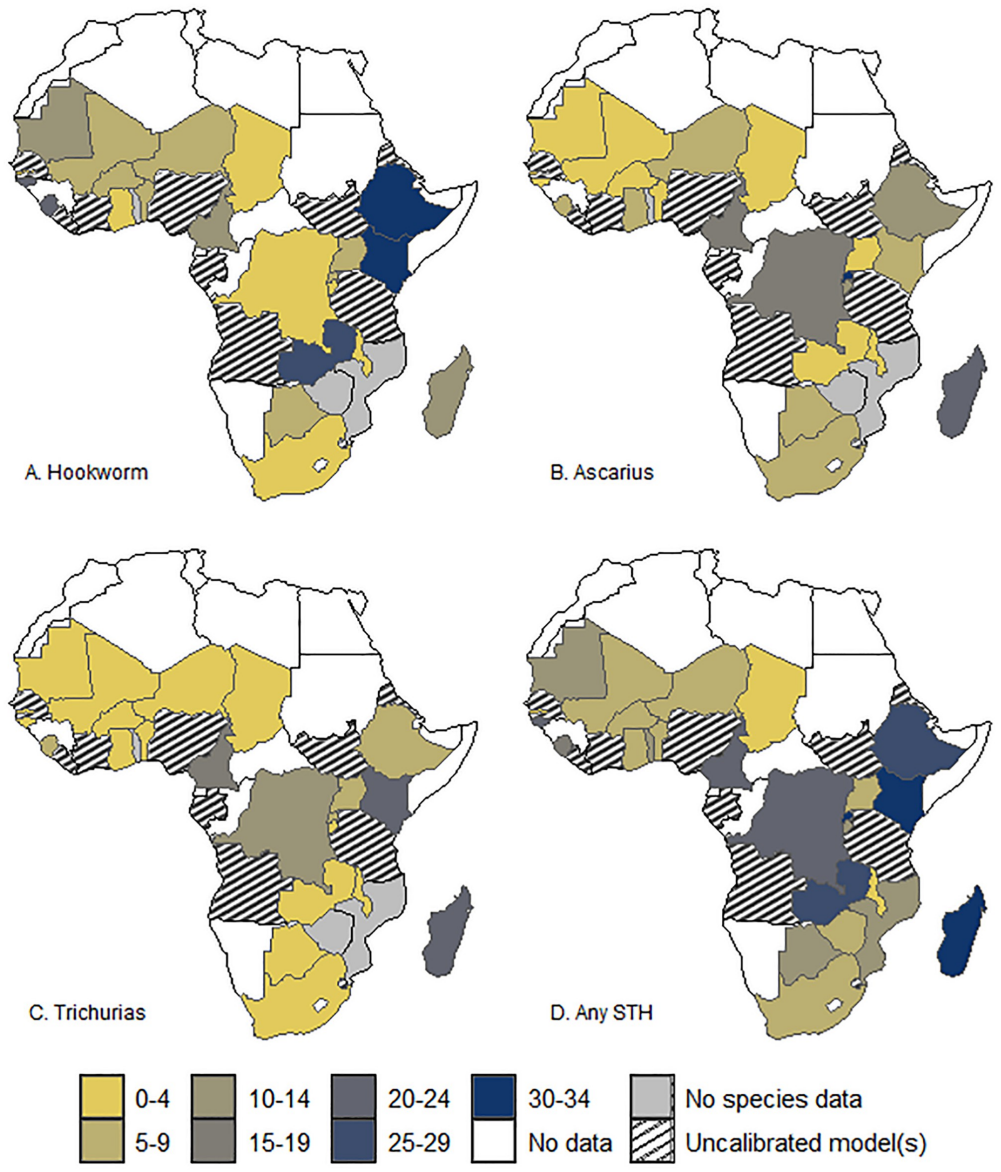
Maps showing the uncertainty (standard deviations) of the country-level predicted prevalence for Hookworm (A), *Ascaris* (B), *Trichiura* (C), and overall STH (D). The map’s boundaries, names, and designations are derived from Global Administrative Areas (GADM), available at https://gadm.org/ [42]. They do not reflect any opinions of the authors or their affiliated institutions regarding the legal status of any country, territory, city, area, or its authorities, nor the delineation of its borders or boundaries.

Fig 3 shows the spatial distribution of the species-specific observed prevalence and overall STH prevalence at the country level in the countries where the models were calibrated. The binomial regression models indicate 11 of the 26 countries with a high prevalence (>20%) of any STH species and overall STH in countries such as Sierra Leone, Mozambique, Rwanda, and Zambia. The figure shows that the highest Hookworm prevalence was observed in the eastern and western parts of Africa. Conversely, the highest *Ascaris* prevalence was observed in southern and eastern Africa. The central and eastern parts of Africa had the highest predicted *Trichiura* prevalence. Overall, the highest prevalence of any STH was in western and eastern Africa, and it was predicted in Sierra Leone, Mozambique, Rwanda, and Zambia. The level of uncertainty, however, varied widely per species and within each country, as seen in the 95% confidence intervals of the estimates (Table 2) and associated uncertainty maps (Fig 4).

**Table 2:** Country-level predicted prevalence estimates and associated 95% confidence intervals.

Country	Year	Hookworm Estimate (95% CI)	*Ascaris* Estimate (95% CI)	*Trichiura* Estimate (95% CI)	Any STH Estimate (95% CI)
**Southern Africa**					
Botswana	2015	5.2% (0.1%,35.4%)	5.6% (0.1%, 34.8%)	1.1% (0.0%,9.9%)	11.4% (0.5%,53.3%)
South Africa	2017	3.2% (0.7%, 8.4%)	27.8% (14.2%,45.2%)	0.1% (0.1%,0.2%)	30.2% (16.6%, 47.3%)
**Central Africa**					
Cameroon	2012	13.0% (0.6%,44.3%)	5.6% (0.0%,66.9%)	8.3% (0.0%,69.9%)	22.9% (0.7%,90.1%)
DRC	2015	1.1% (0.4%, 2.5%)	20.9% (1.3%, 68.6%)	6.3% (0.0%, 58.8%)	26.3% (2.7%,80.6%)
Chad	2015	0.2% (0.0%,1.5%)	0.8% (0.0%,5.2%	0.1% (0.0%,0.4%)	1.1% (0.0%,6.1%)
**Eastern Africa**					
Burundi	2014	4.6% (0.8%,14.2%)	12.5% (1.2%,38.2%)	2.9% (0.2%,12.1%)	18.9% (4.7%,43.8%)
Ethiopia	2009	23.2% (0.0%,97.0%)	10.3% (0.2%,54.1%)	3.0% (0.0%,22.8%)	32.7% (1.1%,97.6%)
Kenya	2015	24.6% (0.0%, 95.4%)	2.6% (0.0%,18.0%)	10.3% (0.0%,87.5%)	33.9% (0.6%,97.9%)
Madagascar	2015	5.3% (0.0%,43.6%)	15.4% (0.1%, 80.6%)	14.2% (0.0%,90.6%)	27.6% (0.6%,97.6%)
Malawi	2018	0.9% (0.1%,3.6%)	1.7% (0.1%,7.3%)	0.1% (0.0%, 0.2%)	2.7% (0.5%, 9.0%)
Mozambique	2007	NA	NA	NA	46.1% (23.0%,70.3%)
Rwanda	2014	4.7% (0.3%, 20.3%)	33.7% (1.1%, 98.8%)	1.8% (0.0%, 18.7%)	38.2% (4.1%, 97.1%)
Uganda	2013	3.5% (0.1%,18.1%)	0.9% (0.0%,6.3%)	2.3% (0.0%,18.3%)	6.5% (0.2%,29.6%)
Zambia	2005	36.0% (1.2%,93.6%)	0.9% (0.0%,4.9%)	0.2% (0.0%,1.0%)	36.7% (1.9%,93.7%)
Zimbabwe	2010	NA	NA	NA	2.5% (0.0%,26.5%)
**Western Africa**					
Benin	2017	9.4% (1.3%,27.5%)	0.9% (0.0%,7.8%)	0.4% (0.3%,0.4%)	10.5% (1.8%,31.2%)
Burkina Faso	2004	3.0% (0.0%,20.3%)	0.0% (0.0%,0.0%)	0.4% (0.2%,0.6%)	3.4% (0.4%,20.7%)
Ghana	2008	2.9% (0.2%,11.8%)	3.3% (0.0%,25.1%)	0.3% (0.0%,1.4%)	6.4% (0.6%, 28.2%)
Guinea-Bissau	2018	26.6% (1.2%,81.5%)	0.1% (0.0%,0.2%)	0.2% (0.1%, 0.4%)	26.8% (1.5%,81.6%)
Mali	2004	2.2% (0.0%,25.9%)	0.0% (0.0%,0.1%)	0.2% (0.0%,0.6%)	2.4% (0.1%, 26.1%)
Mauritania	2015	7.2% (0.1%,49.2%)	1.8% (0.8%,3.4%)	0.7% (0.0%,4.7%)	9.5% (1.5%,50.9%)
Niger	2006	3.3% (0.0%,25.7%)	1.0% (0.0%,8.2%)	0.1% (0.0%,0.4%)	4.3% (0.1%,32.4%)
Sierra Leone	2008	40.9% (9.1%,82.5%)	7.7% (0.8%, 26.0%)	3.3% (0.1%, 18.2%)	47.2% (14.5%, 84.7%)
The Gambia	2015	0.3% (0.1%,1.2%)	0.4% (0.0%,2.3%)	0.1% (0.0%, 0.1%)	0.8% (0.2%, 2.8%)
Togo	2015	NA	NA	NA	12.1% (1.0%,42.2%)

CI = Confidence interval.

DRC = Democratic Republic of the Congo (Congo Kinshasa).

NA = Not available.

The uncertainty maps also illustrate the countries where predictions were produced with low confidence (indicated by high standard deviation, s.d.) and high confidence (indicated by low standard deviation). The levels of uncertainty were generally low (s.d. < 33) for all the species and countries.

Table 2 shows the overall prevalence and confidence intervals for the well-calibrated country models.

#### Geostatistical model parameter estimates at country-level

Variable selection was performed for each country and species. The final selected covariates were utilized to construct predictive geostatistical models specific to each of the three STH species or any STH. In general, there was a negative association between nightlights and all of the species (Table C in S1 Text). Similarly, the amount of soil PH and soil content (silt, sand, or clay) had a negative association with all three species. On the other hand, an increase in the aridity index and precipitation was associated with an increased risk of STH. Furthermore, an increase in the poverty index was associated with an increase in the odds of Hookworm (Table C in S1 Text). The variance and scale of spatial correlation varied extensively by country and species (exponents of coefficients in Figs A-D in S1 Text).

#### Summaries of model validation at country-level

Table 3 shows the summary information on model validation for each country. A country was classified as having an uncalibrated model(s) if the validation for at least one of the hold-out samples in each model did not meet the criteria for being well-calibrated. Overall, 29% (10) of the 35 fitted country-models were uncalibrated in at least one of the holdout samples.

**Table 3:** Summary of model validation analyses per country.

Country	Year	Prevalence (%)				*ϕ* (km)				Calibrated Model (s)
		HK	ASC	TT	Any STH	HK	ASC	TT	Any STH	
**Southern Africa**										
Botswana	2015	55.0	41.0	54.0		391.9	34.2	445.7		Yes
South Africa	2017	24.0	70.0	3.0		384.9	67.7	12.3		Yes
Swaziland	2015	20.0	90.0	70.0		154.4	56.7	263.3		No
**Central Africa**										
Angola	2014	80.0	100.0	43.0		173.1	181.4	69.0		No
Cameroon	2012	60.0	73.0	72.0		94.1	642.2	213.9		Yes
Chad	2015	20.0	36.0	26.0		58.4	134.4	28.7		Yes
DRC	2015	60.0	88.0	94.0		3,027.4	100.4	1,102.2		Yes
Gabon	2015	91.0	100.0	100.0		65.2	8.3	28.4		No
**Eastern Africa**										
Burundi	2014	38.0	70.0	36.0		43.1	49.2	21.3		Yes
Eritrea	2015	4.0	2.0	8.0		61.5	26.9	475.3		No
Ethiopia	2009	75.0	57.0	54.0		32.3	114.5	128.9		Yes
Kenya	2015	50.0	32.0	50.0		52.5	241.7	174.5		Yes
Madagascar	2015	52.0	96.0	98.0		25.9	144.0	169.1		Yes
Malawi	2018	20.0	37.0	7.0		12.3	13.1	11.8		Yes
Mozambique	2007				82				158.2	Yes
Rwanda	2014	44.0	100.0	100.0		21.7	19.8	72.9		Yes
South Sudan	2018	67.0	36.0	29.0		279.9	81.9	268.1		No
Tanzania (Mainland)	2018	50.0	27.0	43.0		78.5	366.2	153.9		No
Uganda	2016	23.0	9.0	12.0		91.8	643.1	202.8		Yes
Zambia	2005	87.0	33.0	12.0		50.7	97.5	157.3		Yes
Zimbabwe	2010				78				50.8	Yes
**Western Africa**										
Benin	2017	45.0	34.0	4.0		90.8	123.0	29.6		Yes
Burkina Faso	2004	75.0	2.0	5.0		107.1	19.6	716.2		Yes
Cote d’Ivoire	2014	78.0	56.0	74.0		98.0	626.2	88.0		No
The Gambia	2015	12.0	60.0	8.0		44.8	11.0	4.9		Yes
Ghana	2008	27.0	20.0	5.0		141.8	32.9	120.5		Yes
Guinea-Bissau	2018	100.0	4.0	12.0		21.6	26.6	1.1		Yes
Liberia	2015	42.0	100.0	16.0		94.6	19.8	91.6		No
Mali	2004	100.0	6.0	7.0		104.1	370.9	229.1		Yes
Mauritania	2015	100.0	8.0	10.0		68.9	92.1	915.6		Yes
Niger	2006	5.0	27.0	8.0		193.5	67.5	1,316.5		Yes
Nigeria	2014	86.0	94.0	77.0		112.8	58.5	228.3		No
Senegal	2013	61.0	64.0	78.0		43.3	34.9	43.1		No
Sierra Leone	2008	95.0	25.0	30.0		43.9	22.3	24.0		Yes
Togo	2015				100				25.8	Yes

HK = Hookworm, ASC = *Ascaris*, TT = *Trichiura*.

*ϕ* = Estimated scale of spatial correlation.

DRC = Democratic Republic of the Congo (Congo Kinshasa).

### Country example: Rwanda

#### Predicted prevalence of STH in Rwanda

**Fig 5:**
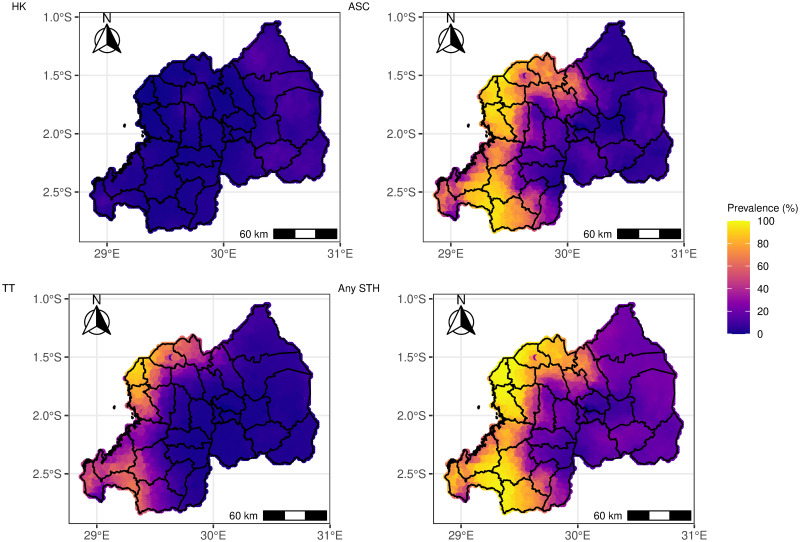
Map showing the pixel-level predicted geographic distribution of the prevalence of STH in Rwanda (HK = Hookworm, ASC = *Ascaris*, TT = *Trichiura* and Any STH = Overall STH). The map’s boundaries, names, and designations are derived from Global Administrative Areas (GADM), available at https://gadm.org/ [42]. They do not reflect any opinions of the authors or their affiliated institutions regarding the legal status of any country, territory, city, area, or its authorities, nor the delineation of its borders or boundaries.

**Fig 6:**
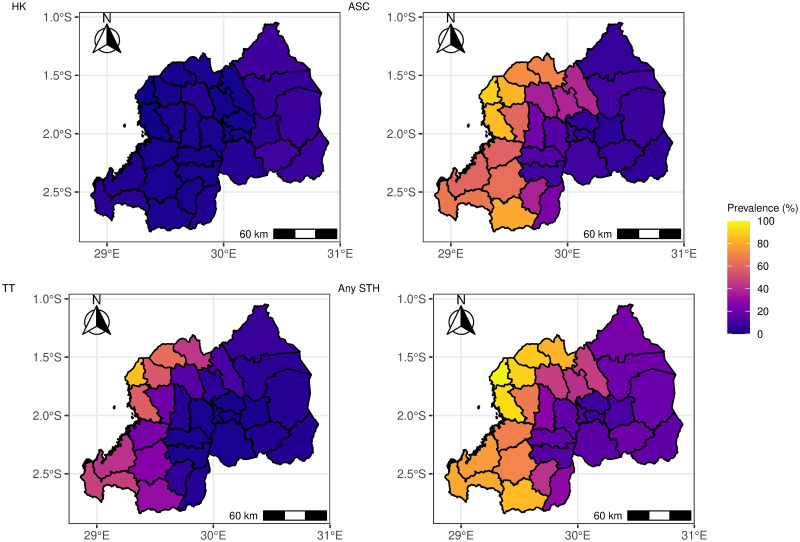
Map showing the subnational-level predicted geographic distribution of the prevalence of STH in Rwanda (HK = Hookworm, ASC = *Ascaris*, TT = *Trichiura* and Any STH = Overall STH). The map’s boundaries, names, and designations are derived from Global Administrative Areas (GADM), available at https://gadm.org/ [42]. They do not reflect any opinions of the authors or their affiliated institutions regarding the legal status of any country, territory, city, area, or its authorities, nor the delineation of its borders or boundaries.

The predicted point prevalence of both STH species and overall STH in Rwanda are presented in Fig 5. Overall, the predicted prevalence of any STH species and any STH is heterogeneously distributed across Rwanda. A notably heightened burden of STH infections was documented in the western regions of Rwanda, with *Ascaris* demonstrating the highest prevalence, closely followed by *Trichiura* (Fig 5). These findings are also evident in the sub-national predicted prevalence maps (Fig 6). The confidence intervals for both the point and sub-national prevalence maps are given in the Shiny application.

#### Point and exceedance probability maps of soil-transmitted helminths in Rwanda

**Fig 7:**
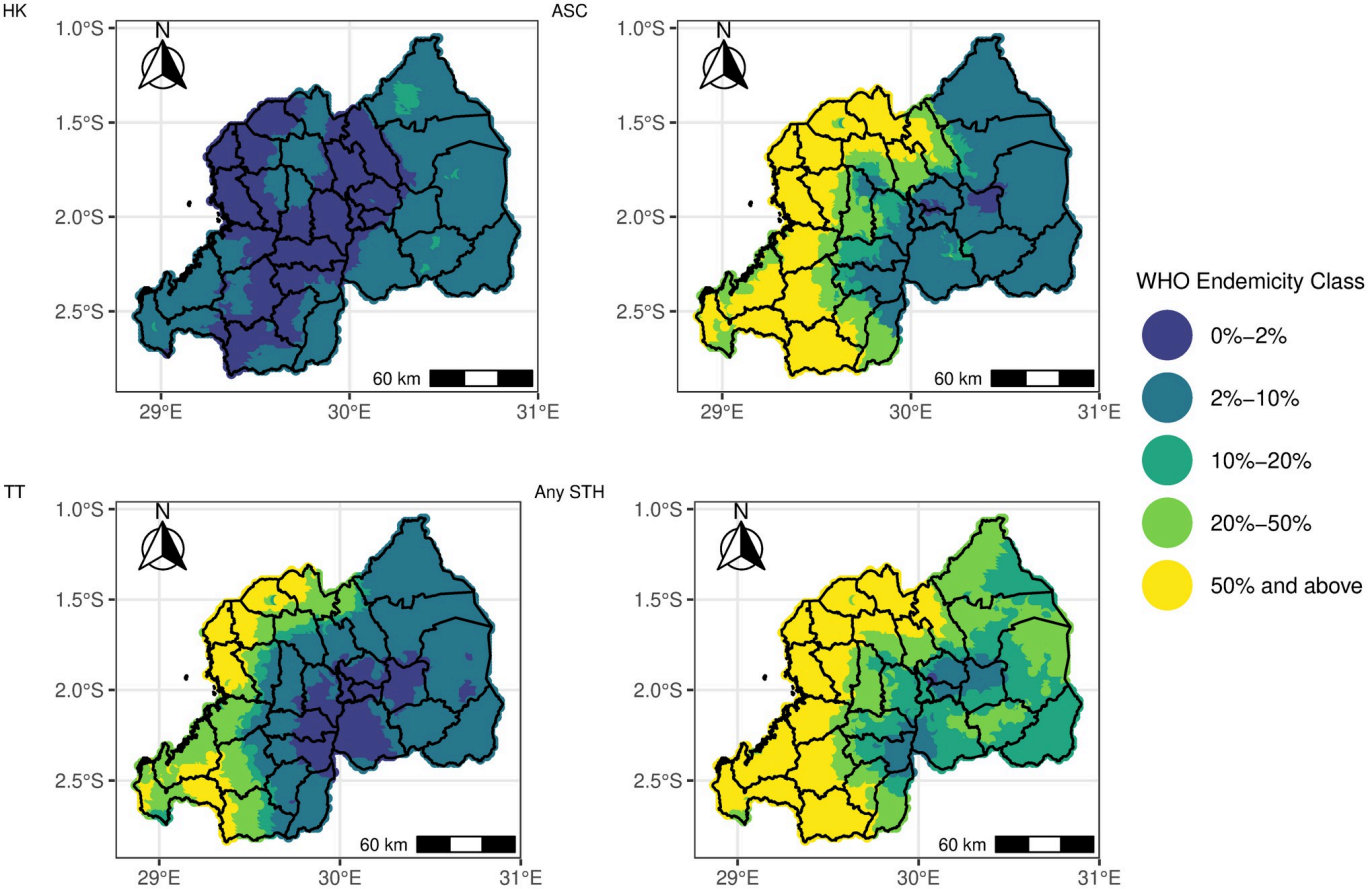
Map showing the predicted STH (HK = Hookworm, ASC = *Ascaris*, TT = *Trichiura*, STH = any STH) endemicity class in Rwanda at the pixel level from the Binomial regression model in 2. The map’s boundaries, names, and designations are derived from Global Administrative Areas (GADM), available at https://gadm.org/ [42]. They do not reflect any opinions of the authors or their affiliated institutions regarding the legal status of any country, territory, city, area, or its authorities, nor the delineation of its borders or boundaries.

**Fig 8:**
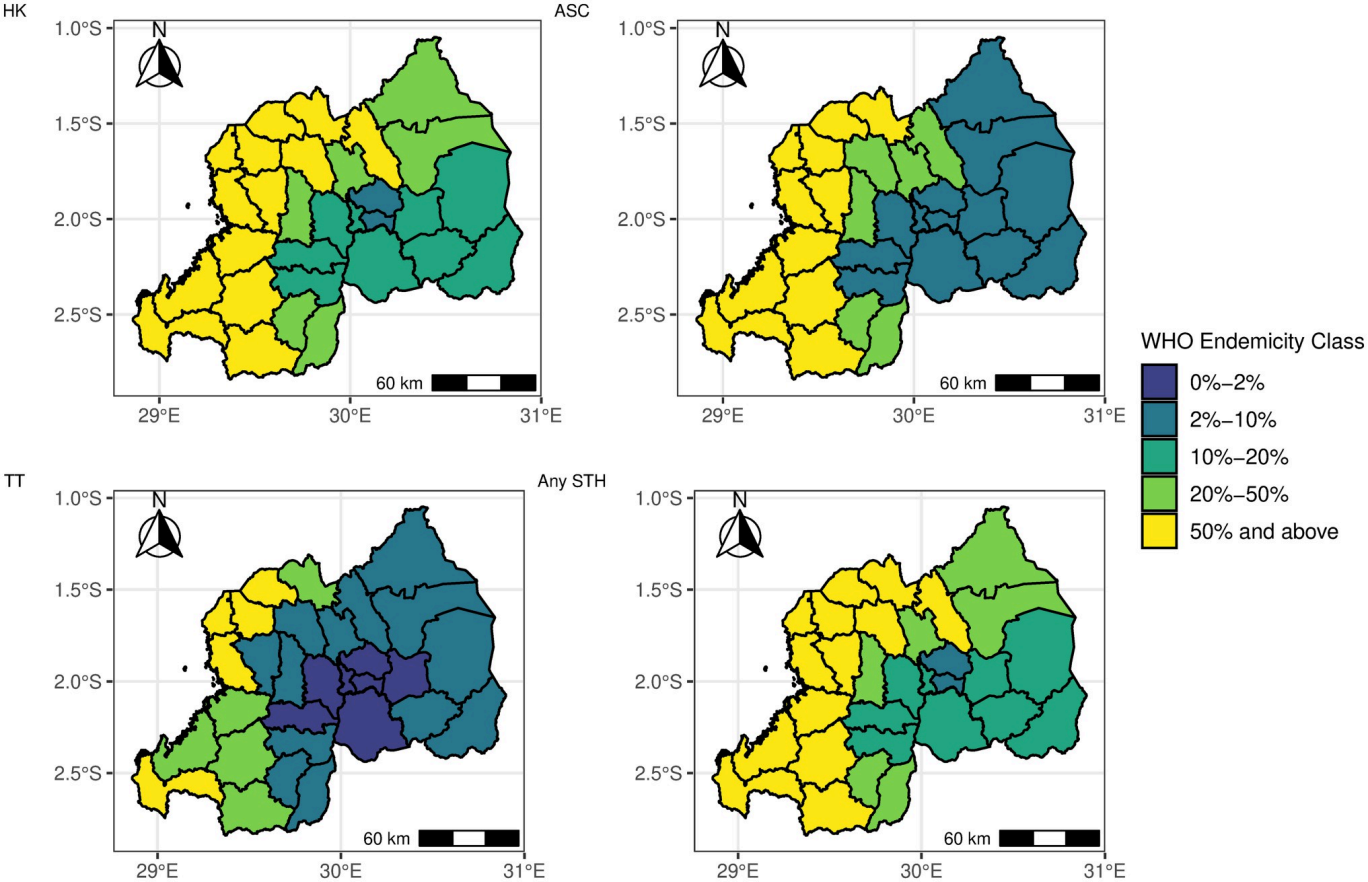
Map showing the predicted STH (HK = Hookworm, ASC = *Ascaris*, TT = *Trichiura*, STH = any STH) endemicity class in Rwanda at the subnational level from the Binomial regression model in 2. The map’s boundaries, names, and designations are derived from Global Administrative Areas (GADM), available at https://gadm.org/ [42]. They do not reflect any opinions of the authors or their affiliated institutions regarding the legal status of any country, territory, city, area, or its authorities, nor the delineation of its borders or boundaries.

The binomial regression models indicate a lot of areas with a high prevalence (>20%) of any STH species and any STH in Rwanda. Figs 7 and 8 show the WHO predicted endemicity class STH treatment at pixel and sub-national levels. The maps depict high exceedance probabilities in the central and the western sides of Rwanda. These are, therefore, the treatment priority areas for STH.

#### Geostatistical model parameter estimates for Rwanda

The modeling suggests a strong relationship between rainfall and *Ascaris* and *Trichiura* in Rwanda (Table 4). An increase in the amount of rainfall was seen to increase the prevalence of the two species. Conversely, soil content (sand, silt, clay) was associated with a reduction in the prevalence of all STH species. Likewise, an increase in nightlights was associated with a reduction in the prevalence of *Ascaris* and *Trichiura*.

**Table 4:** Monte Carlo maximum likelihood estimates and associated 95% confidence intervals for the model in Eq 2 for Rwanda.

Parameter	Hookworm Estimate (95% CI)	*Ascaris* Estimate (95% CI)	*Trichiura* Estimate (95% CI)
Intercept	-1.555 (-2.739, -0.372)	-6.153 (-6.883, -5.422)	-5.307 (-7.481, -3.133)
Soil	-0.007 (-0.012, -0.001)	-0.008 (-0.010, -0.006)	-0.005 (-0.007, -0.002)
Precipitation	NA	0.096 (0.092, 0.101)	0.064 (0.039, 0.090)
Nightlights	NA	-0.168 (-0.227, -0.108)	-0.259 (-0.420, -0.100)
*σ* ^2^	1.131 (0.529,2.420)	1.441 (0.858,2.418)	1.750 (0.459, 6.670)
*ϕ*	21.727 (8.313, 56.785)	19.774 (8.972,43.584)	72.933 (14.913,356.672)

CI = Confidence interval.

Soil = sand, clay, or silt content.

NA corresponds to the situation where the covariate was not included in the model.

*σ*^2^ = Estimated variance; *ϕ* = Estimated scale of spatial correlation.

Table 4 also shows the differences in the covariance parameters for the three species. The point estimates for the scale parameter were 21.73 km (Hookworm), 19.77 km (*Ascaris*), and 72.91 km (*Trichiura*).

#### Model validation for Rwanda

**Fig 9:**
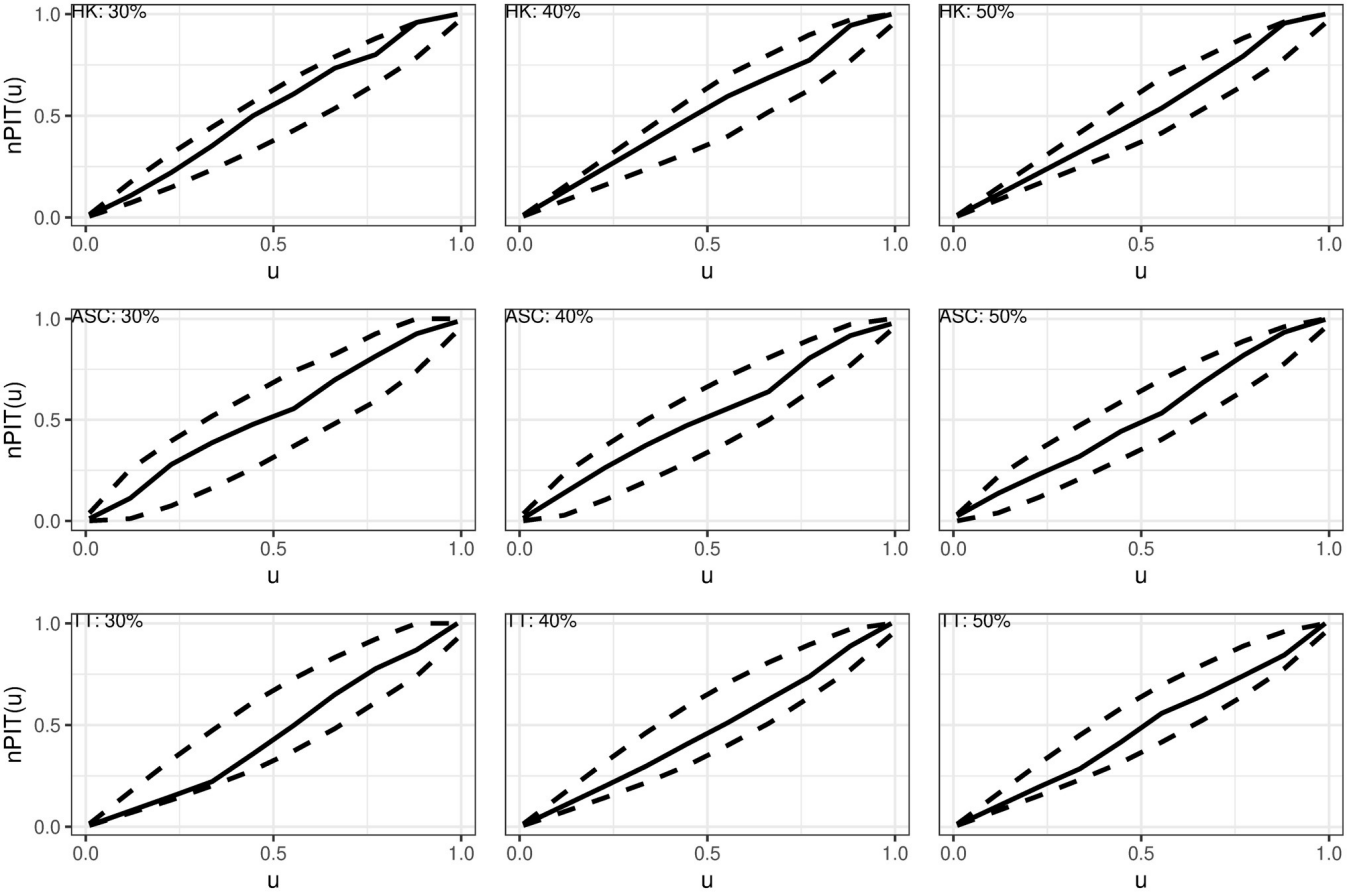
Plots of the non-randomized probability integral transform (nrPIT) calculated for three (30%, 40%, 50%) hold-out samples for Hookworm (HK), *Ascaris* (ASC), and *Trichiura* (TT).

Fig 9 illustrates the model validation plots for the Rwanda models. The figure shows that the observed nrPIT curves (represented by the solid black line) from the three hold-out samples for all three species fall within the 95% envelope (denoted by the dashed lines). We, therefore, conclude that we do not have enough evidence to reject the null hypothesis of well-calibrated models.

## Discussion

In this study, we have carried out a comprehensive geostatistical analysis of soil-transmitted infections data from the ESPEN database. We developed geostatistical models separately for each country, so as to tailor the selection of spatial covariates and estimation of covariance parameters to the heterogeneous spatial patterns across countries. In countries where the geostatistical models were validated successfully, we proceeded to generate predictions of STH prevalence at both national and sub-national levels.

The selection of covariates used to assist in the geostatistical prediction of prevalence showed different results across countries. However, notably, due to the weak empirical strength of association with disease prevalence, only a few covariates were selected for most countries. The low predictive power of the spatial covariates may be attributed to the relatively low prevalence levels that are observed in most countries, which make the estimation of regression relationships more cumbersome. Despite these challenges, where predictors were included, they provided some comparable estimates with findings from previous studies. For instance, areas with increased precipitation were associated with a higher likelihood of all STH species, consistent with existing research indicating higher prevalence in wetter regions [3, 30, 31, 52]. Similarly, the observation that an increased amount of nightlights, serving as a proxy for wealth status, decreased the likelihood of all STH species aligns with the established notion of higher prevalence in economically disadvantaged areas [3, 29]. Additionally, the finding that soil pH and content (sand, silt, clay) reduced the likelihood of STH is also consistent with previous research findings [3, 28, 52, 53].

The analysis reveals significant heterogeneity in the estimates of the scale of spatial correlation and the variance of residual spatial variation across countries. The scale of spatial correlation ranged from 1.14 km to 3,027.44 km, while the variance ranged from 0.02 to 95.01 across the countries. Therefore, the wide variations in the estimates of spatial correlation across countries, coupled with observed non-stationarity, further justify the use of species-specific, single-country models for this STH data. The non-stationarity is likely driven by differing control intervention histories across countries, which are challenging to capture adequately using the available spatial covariates. These intervention histories can significantly influence the spatial distribution and prevalence of STH, leading to localized variations that a global model might fail to account for.

Moreover, it was observed that the geostatistical models exhibited inadequate calibration in certain countries, prohibiting spatial predictions at unsampled locations. This issue may be attributed to a combined effect of very sparse data and small estimated spatial correlations relative to the study area. For some countries where the estimated variance of the residual spatial process is relatively small, an additional explanation for the poor calibration of the geostatistical models might be the presence of strong noise components that diminish the spatial signal within the data. These findings consequently urge caution in developing an Africa-wide model based solely on ESPEN data, given the observed heterogeneity in the model parameter estimates and the challenges encountered in model calibration across different regions and species.

In our study, we used data for a single time point for all the countries, namely the most recent survey. Hence, one of the main limitations is the absence of a spatio-temporal geostatistical model that could make full use of all the historical data. However, the availability of data over time varies from country to country, with some countries providing only a single survey. The average number of surveys per country was 6, with the minimum being 1 survey and the maximum being 15 surveys per country. An additional challenge in building credible spatio-temporal models for STH is the effect on prevalence trends due to mass drug administration (MDA). Information on the frequency and coverage of MDA is an essential element that should be incorporated in such models; however, not all countries provide this information at suitable spatial and temporal resolutions for geostatistical models. Future research should aim to bridge geostatistical models with mathematical models capable of integrating MDA data, offering a valuable approach for combining information from baseline to impact surveys.

## Conclusion

This study demonstrates the use of model-based geostatistics to harness ESPEN data, offering valuable insights into the spatial distribution of STH prevalence across countries. While ESPEN data serve as a crucial resource for understanding spatial patterns in STH prevalence through geostatistical models, inherent limitations arise from the sparsity of data, both temporally and spatially in certain countries, constraining the applicability of such models. Nevertheless, the predictive inferences derived from these models, where possible, provide useful information for national control programs, facilitating targeted interventions and informing survey designs for future STH assessments.

## Supporting information

S1 Text Supporting information. (PDF)
