# Supplementary material for: Using ESPEN data for evidence-based control of neglected tropical diseases in sub-Saharan Africa: A comprehensive model-based geostatistical analysis of soil-transmitted helminths
Source: PLoS Negl Trop Dis. 2025 Jan 9;19(1):e0012782. doi: 10.1371/journal.pntd.0012782 (PMC11753640; doi:10.1371/journal.pntd.0012782)
Supplement: S1 Text — (PDF) [file pntd.0012782.s001.pdf]

Using ESPEN Data for Evidence-Based Control of Neglected Tropical Diseases in sub-Saharan Africa: a Comprehensive Model-based Geostatistical Analysis of Soil-Transmitted Helminths

Supporting information

1 Summary of Data Characteristics: Countries, Dates, and Sample Sizes

Table A outlines the countries included in the study, the sample sizes, and the years the data were collected. The sample sizes are the raw data obtained from the ESPEN website, prior to merging the geolocated STH data with the spatial covariates.

Table A. Countries included in the analysis, data collection dates, and sample sizes per country.

Country	Year	Sample size	Mean (Std. dev)	Median (IQR)
**Southern Africa**				
Botswana	2015	128	46 (7)	48 (43, 50)
South Africa	2017	152	41 (11)	47 (35, 49)
Swaziland	2015	262	50 (3)	50 (50, 50)
**Central Africa**				
Angola	2014	121	29 (3)	30 (30, 30)
Cameroon	2012	184	50 (0)	50 (50, 50)
Chad	2015	281	49 (4)	50 (50, 50)
DRC	2015	112	57 (22)	50 (50, 50)
Gabon	2015	182	49 (25)	48 (34, 53)
**Eastern Africa**				
Burundi	2014	209	50 (0)	50 (50, 50)
Eritrea	2015	162	51 (8)	52 (50, 56)
Ethiopia	2009	102	105 (32)	105 (97, 107)
Kenya	2015	63	58 (4)	58 (56, 60)
Madagascar	2015	305	51 (10)	50 (50, 50)
Malawi	2018	277	29 (2)	30 (29, 30)
Mozambique	2007	134	50 (0)	50 (50, 50)
Rwanda	2014	183	50 (1)	50 (50, 50)
South Sudan	2018	103	48 (4)	50 (49, 50)
Tanzania (Mainland)	2018	301	34 (10)	30 (30, 30)
Uganda	2013	83	58 (14)	60 (60, 63)
Zambia	2005	57	60 (4)	60 (60, 61)
Zimbabwe	2010	126	42 (10)	45 (37, 49)
**Western Africa**				
Benin	2017	66	70 (31)	50 (49, 80)
Burkina Faso	2004	87	59 (6)	60 (59, 61)
Cote d'Ivoire	2014	529	31 (2)	30 (30, 32)
The Gambia	2015	206	50 (5)	50 (49, 50)
Ghana	2008	76	59 (4)	60 (60, 60)
Guinea-Bissau	2018	55	49 (2)	50 (50, 50)
Liberia	2015	320	49 (4)	50 (50, 50)
Mali	2004	187	71 (18)	69 (66, 70)
Mauritania	2015	72	57 (39)	50 (47, 62)
Niger	2006	73	65 (31)	60 (60, 60)
Nigeria	2014	705	50 (5)	50 (50, 52)
Senegal	2013	117	50 (5)	50 (50, 50)
Sierra Leone	2008	114	83 (32)	99 (55, 110)
Togo	2015	1077	15 (0)	15 (15, 15)

Std. dev = Standard deviation.
IQR = Interquartile range (25th quartile, 75th quartile).

Table B outlines the countries excluded from the study, the sample sizes, and the years the data were collected.

Table B. Countries excluded from the analysis, data collection dates, and sample sizes per country.

**Country**	**Year**	**Sample size**	**Comment**
Algeria	NA	NA	No data (zero data points) for STH
Cape Verde	2012	9	All data points do not have location data
Central African Republic	1983	1	None
Comoros	NA	NA	No data (zero data points) for STH
Equatorial Guinea	NA	NA	No data (zero data points) for STH
Guinea	2013	40	None
Mauritius	2015	47	None
Republic of Congo	1985	1	None
Seychelles	2014	6	None
Tanzania (Zanzibar)	2011	40	None

NA = Not available.

2 Non-randomized probability integral transform for binomial geostatistical models

This supplementary material gives detailed information on the non-randomized probability integral transform (nrPIT) as outlined by Czado et al. and modified by Giorgi et al. for binomial geostatistical models and [1, 2].

Let $Y = \{Y_i; 1 =, \ldots, n\}$ denote the vector of random variables of the number of STH (any STH or species-specific) positive cases, Y_i out of n_i tested individuals at location x_i, for $i = 1, \ldots, n$. We assume that Y_i follows a Binomial distribution with probability $p(\mathbf{x}_i)$ and linear predictor

$$\log\left\{\frac{p(\mathbf{x}_i)}{1 - p(\mathbf{x}_i)}\right\} = d(\mathbf{x}_i)^\top \beta + S(\mathbf{x}_i) + Z_i,$$

where β is the vector of regression coefficients associated with the matrix of covariates $d(\mathbf{x}_i)$. $S(\mathbf{x}_i)$ and Z_i are the Gaussian process and Gaussian noise, respectively, that have a mean of zero and variance of σ^2 and τ^2.

To outline the nrPIT we let $Q(Z)$ denote the cumulative density function of a random variable Z and Y_i^* denote the random variable of the positive tested STH (any STH or species-specific) cases at a set of hold-out locations say $\mathbf{x}_j^*$ for $j =, \ldots, q$. Conditional on $Y = y$, the conditional cumulative probability distribution (CPD) of Y_i^* is given as:

$$Q(y_j^*|y) = P(Y_j^* \leq y_j^*|y_1, \ldots, y_n). \tag{1}$$

To compute Eq 1, we first define $W = \{S(\mathbf{x}_i) + Z_i : i = 1, \ldots, n\}$ and $W_j^* = S(\mathbf{x}_j^*) + Z_j$ for $j = 1, \ldots, q$. Since it follows from the model assumptions that $Q(y_j^*|w_j, y) = Q(y_j^*|w_j)$, Eq 1 is expressed as:

$$\begin{aligned} Q(y_j^* \mid y) &= \int_{-\infty}^{+\infty} f(w_j \mid y) Q(y_j^*|w_j, y)\, dw_j \\ &= \int_{-\infty}^{+\infty} f(w_j \mid y) Q(y_j^* \mid w_j)\, dw_j \end{aligned} \tag{2}$$

where $Q(y_j^*|w_j)$ is the CPD of a Binomial distribution with number of trials n_j and

probability $p(x_j^*)$ and $f(w_j|y)$ is the density function of the predictive distribution of W_j. To compute the integral in Eq 2, we then simulated $10,000$ samples from $f(w_j|y)$ as follows: 1) simulate $10,000$ samples from W conditionally on y; 2) use the resulting samples from the previous step to simulate from W_j given W, which corresponds to a multivariate Gaussian distribution. More details on this can be found in other texts [3].

Consequently, the nrPIT is defined as

$$\text{nrPIT}(u \mid y_j^*, y) = \begin{cases} 0 & \text{if } u \leq Q(y_j^* - 1 \mid y) \\ \frac{[u - Q(y_j^* - 1 \mid y)]}{[Q(y_j^* \mid y) - Q(y_j^* - 1 \mid y)]} & \text{if } Q(y_j^* - 1 \mid y) \leq u \leq Q(y_j^* \mid y) \cdot \\ 1 & \text{if } u \geq Q(y_j^* \mid y) \end{cases} \quad (3)$$

We evaluate the calibration of the model by computing the average nrPIT across all counts. This is expressed as:

$$\text{nrPIT}(u) = \frac{1}{q} \sum_{j=1}^{q} \text{nrPIT}(u \mid y_j^*, y). \quad (4)$$

Czado et al demonstrated, assuming a well-calibrated model, that the expected value of $nrPIT(u)$ is u under the assumption of a well-calibrated model [1].

To perform the diagnostic assessment for our models, we randomly choose three subsets of locations, representing 30%, 40%, and 50% of the data-sets under consideration. The count of positive cases in these hold-out sets is denoted as yj in the aforementioned equations.

We generated a plot of nrPIT($j/10$) against $j/10$ for $j = 1, \ldots, 10$ to evaluate the calibration of the models. We also generated a 95% confidence envelope using the following steps: 1) simulating 10,000 Binomial observations from the distribution of y_j^*, given $W_j = w_j$, where w_j is determined as outlined in our approximation of Eq 4; 2) for each simulated Binomial dataset, calculate the nrPIT as defined in Eq 4; 3) utilizing the resulting 10,000 nrPITs to compute 95% confidence intervals for nrPIT($j/10$) at $j = 1, \ldots, 10$. This process ensures that the 95% confidence intervals are generated under the "null hypothesis" of a well-calibrated model.

3 Spatial covariate parameter estimates for country-level models

4 Estimates of log scale of spatial correlation from geostatistical models

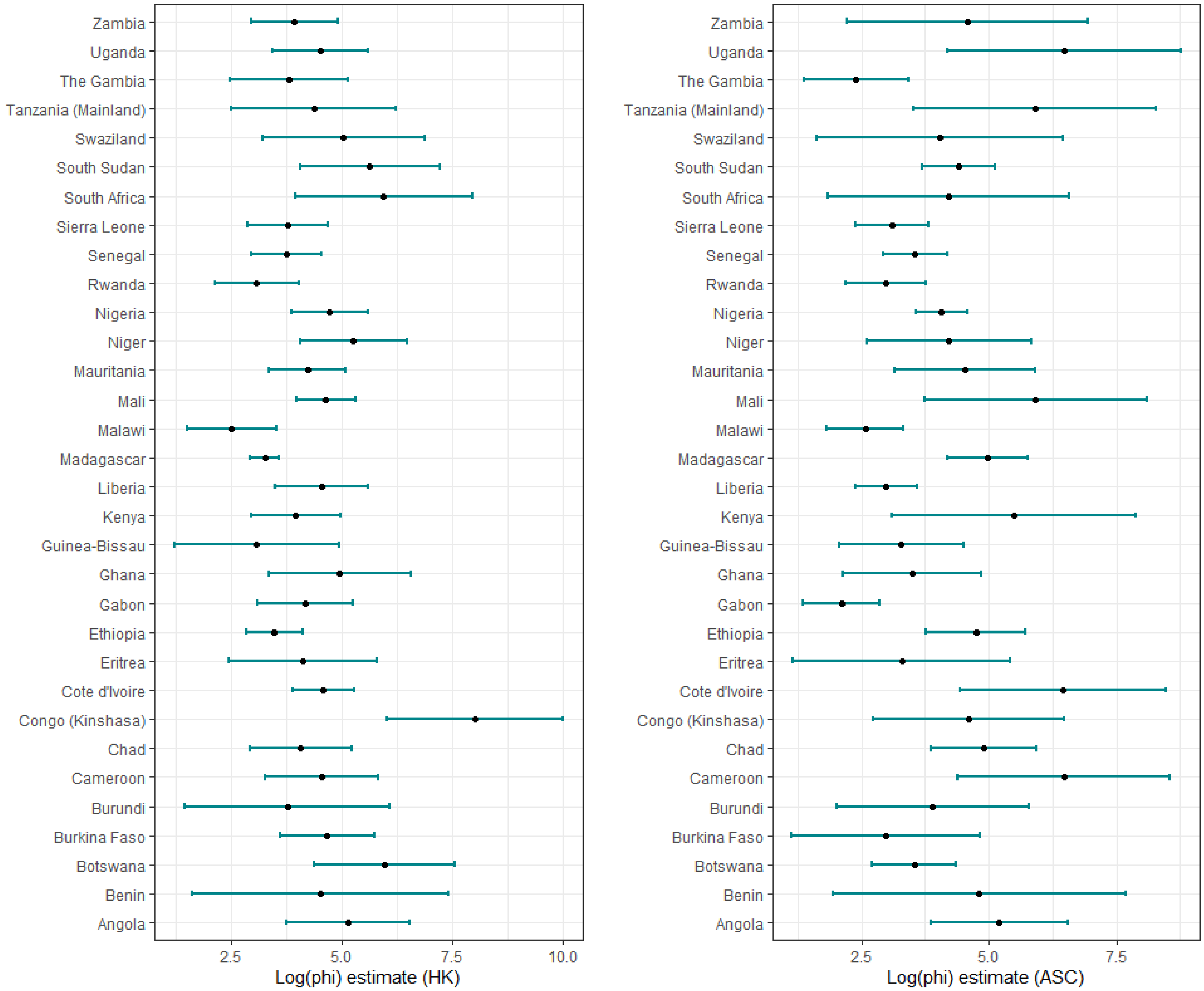

Fig A. Graph showing the estimated log of the scale of the spatial correlation per country for Hookworm (HK) and Ascaris (ASC)).

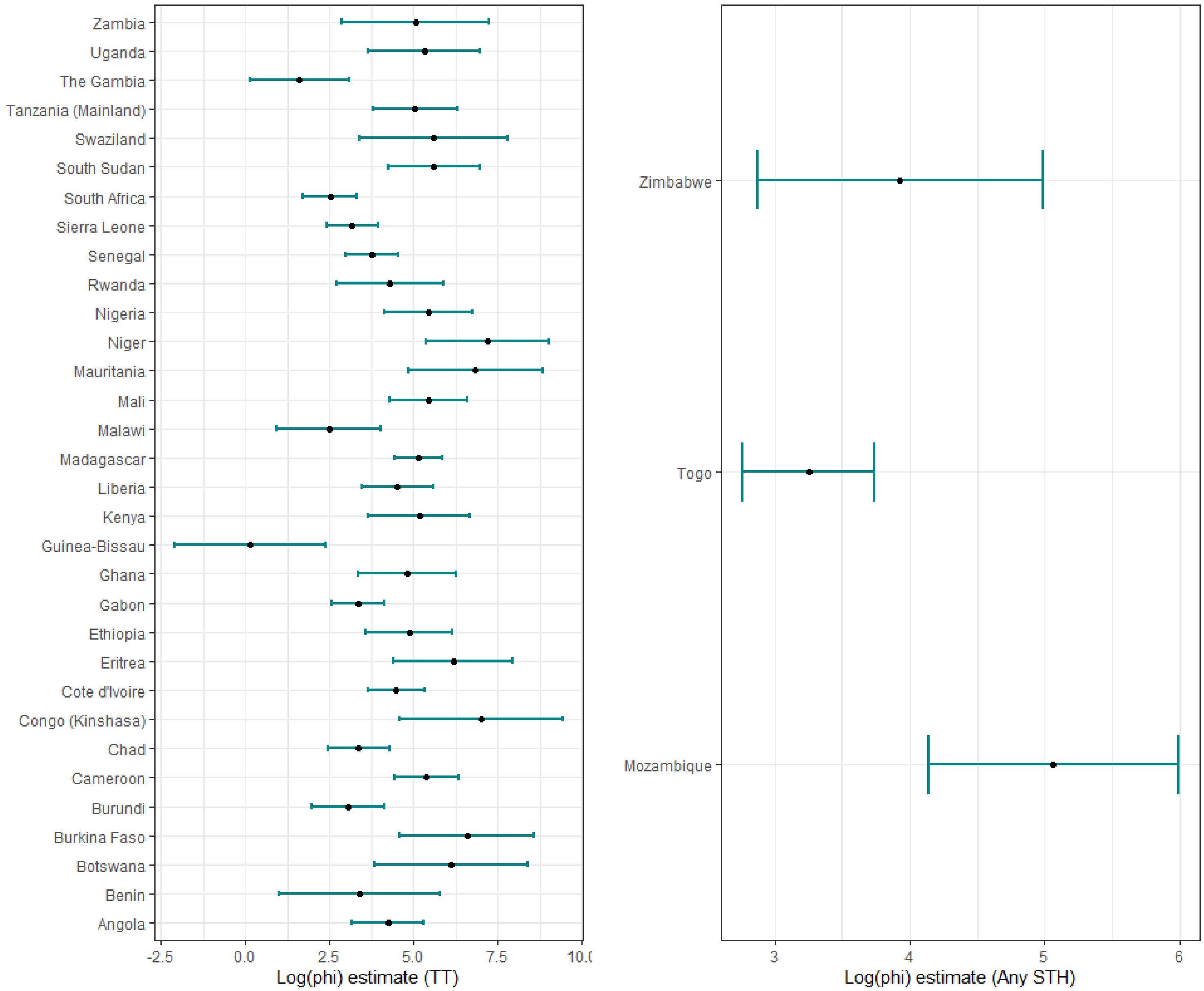

Fig B. Graph showing the estimated log of the scale of the spatial correlation per country for Trichuria (TT) and any STH (STH)).

Table C. Summary of Monte Carlo maximum likelihood estimates of geostatistical models for all country models.

Country	Variable parameter estimate direction															
	Hookworm				Ascaris				Trichiura				Any STH			
	1	2	3	4	1	2	3	4	1	2	3	4	1	2	3	4
**Southern Africa**																
Botswana	-	N	+	N	N	N	+	N	-	N	+	N				
South Africa	N	N	N	N	N	N	N	N	N	N	N	N				
Swaziland	N	N	+	N	N	N	+	N	N	N	N	N				
**Central Africa**																
Angola	-	N	+	N	N	N	N	-	N	N	N	N				
Cameroon	-	N	+	-	N	N	N	N	N	N	N	-				
Chad	N	N	+	-	N	N	N	-	N	N	N	N				
DRC	N	N	N	N	N	N	+	-	N	N	+	-				
Gabon	-	N	+	-	-	N	+	N	-	N	+	-				
**Eastern Africa**																
Burundi	N	N	N		N	N	N	-	N	N	N	-				
Eritrea	N	N	+	N	N	N	N	N	N	N	N	N				
Ethiopia	-	N	-	N	N	N	N	N	N	N	N	-				
Kenya	N	+	N	N	N	N	+	N	N	N	N	N				
Madagascar	-	N	+	N	N	N	+	N	N	N	+	N				
Malawi	N	N	N	-	N	N	N	N	N	N	N	N				
Mozambique													N	N	+	N
Rwanda	N	N	N	-	-	N	+	-	-	N	+	-				
South Sudan	N	N	+	-	N	N	+	-	N	N	N	N				
Tanzania Mainland	N	N	+	N	N	N	+	N	N	N	+	N				
Uganda	N	N	N	N	N	N	N	N	N	N	N	N				
Zambia	N	N	+	N	N	N	N	N	N	N	N	N				
Zimbabwe													N	N	+	N
**Western Africa**																
Benin	-	N	N	N	N	N	N	N	N	N	N	N				
Burkina Faso	N	N	+	N	N	N	N	N	N	N	N	N				
Cote d'Ivoire	-	N	N	-	N	N	N	N	-	N	+	-				
Ghana	N	N	N	N	N	N	N	N	N	N	N	N				
Guinea-Bissau	-	N	+	N	N	N	N	N	N	N	N	N				
Liberia	N	N	N	N	N	N	N	N	-	N	N	N				
Mali	-	N	+	N	N	N	N	N	N	N	N	N				
Mauritania	N	N	+	N	N	N	N	N	N	N	N	N				
Niger	N	N	+	N	N	N	N	N	N	N	N	N				
Nigeria	N	+	+	-	N	N	+	N	N	N	+	-				
Senegal	N	N	+	N	-	N	N	N	N	N	N					
Sierra Leone	N	N	+	N	N	N	+	N	N	N	+	-				
Togo													-	N	N	N

Covariate 1 = Precipitation; 2 = Poverty index; 3 = Precipitation or Aridity index; 4 = Soil type (clay, sand, or silt) or soil PH.
+ = Positive association; - = Negative association; N = Not included in the model.
DRC = Democratic Republic of the Congo (Congo Kinshasa).

5 Estimates of variance of spatial correlation from geostatistical models

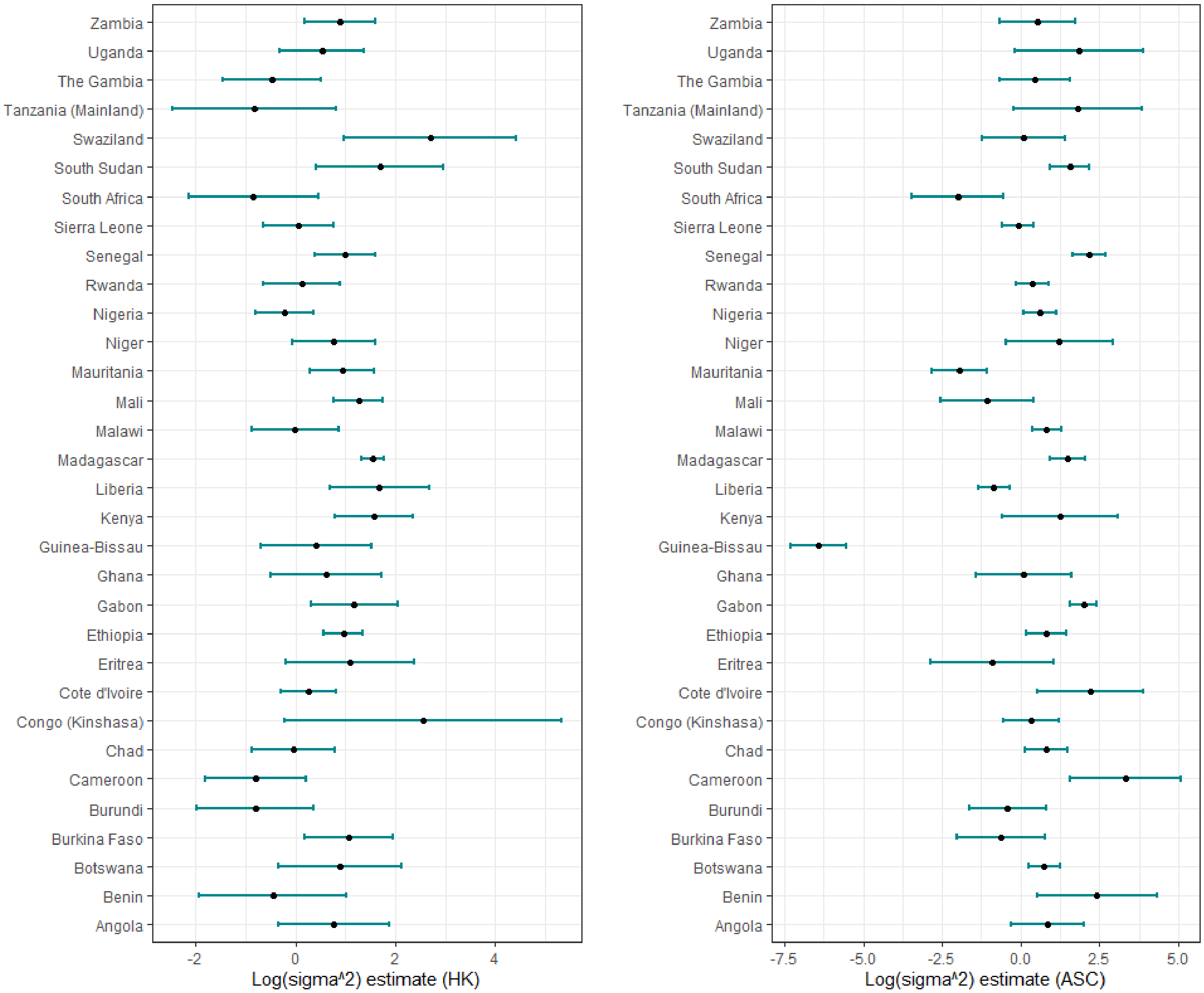

Fig C. Graph showing the estimated log of variance of spatial correlation per country for Hookworm (HK) and Ascaris (ASC)).

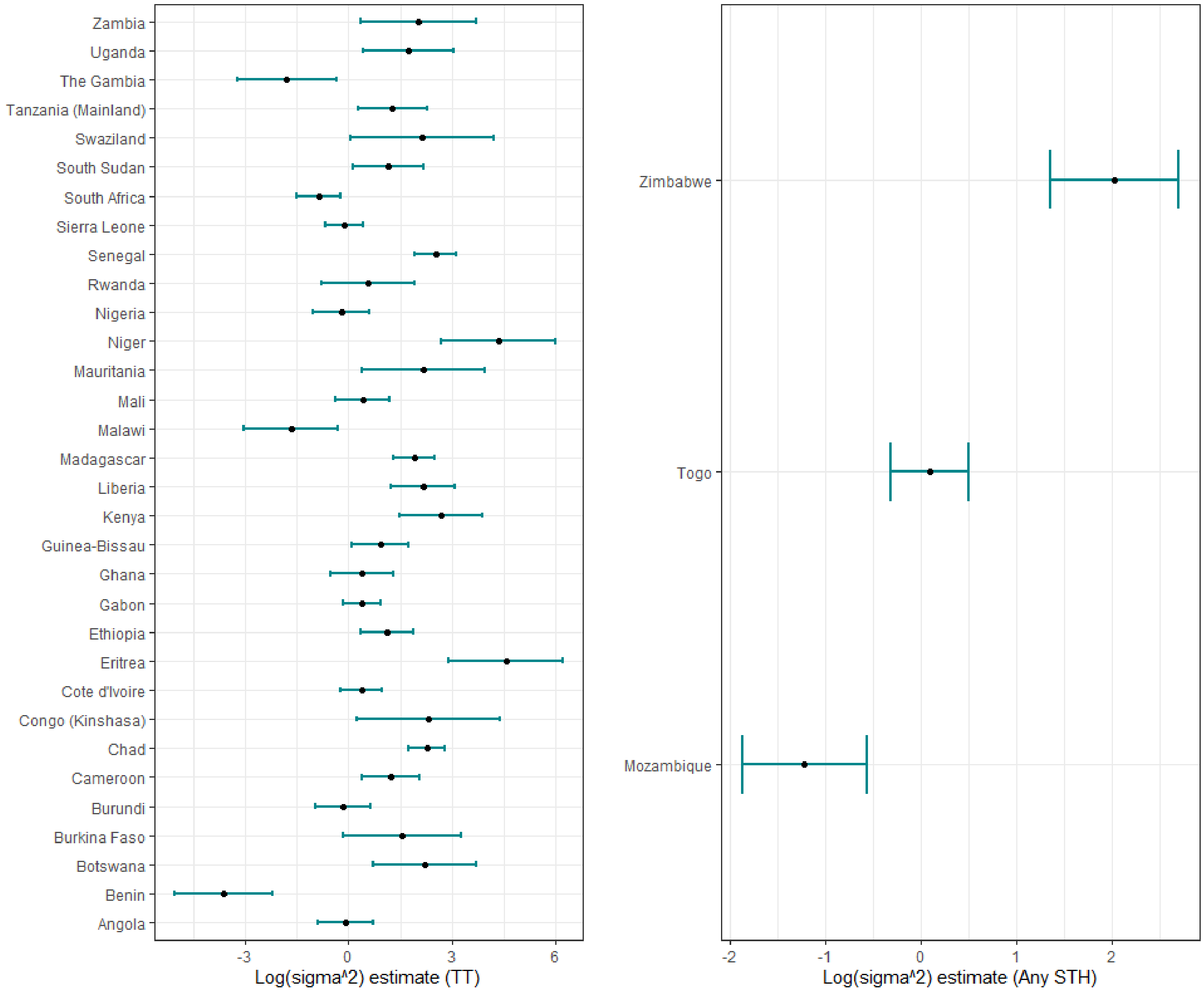

Fig D. Graph showing the estimated log of variance of spatial correlation per country for Trichuria (TT) and any STH (STH)).

References

1. Czado, Claudia and Gneiting, Tilmann and Held, Leonhard. Predictive model assessment for count data. Biometrics. 2009; 65(4):1254-1261.

2. Giorgi, Emanuele and Fronterrè, Claudio and Macharia, Peter M and Alegana, Victor A and Snow, Robert W and Diggle, Peter J. Model building and assessment of the impact of covariates for disease prevalence mapping in low-resource settings: to explain and to predict. The Journal of the Royal Society Interface. 2021; 18(179):202110104.

3. Giorgi, Emanuele and Diggle, Peter J. PrevMap: an R package for prevalence mapping. Journal of Statistical Software. 2017; 78:1–29.
